# Climate security dialogues on Twitter: An annotated dataset

**DOI:** 10.1016/j.dib.2024.110628

**Published:** 2024-06-12

**Authors:** Bia Carneiro, Giulia Tucci

**Affiliations:** a Bioversity International, Via di San Domenico 1, 00153 Rome, Italy; b School of Communication, Federal University of Rio de Janeiro (UFRJ). Av. Pasteur, 250 - Praia Vermelha, Rio de Janeiro, 22290-240, Brazil; c International Center for Tropical Agriculture (CIAT), Via di San Domenico 1, 00153 Rome, Italy

**Keywords:** Social network analysis, Hashtags, Social media, Sentiment analysis, Text mining

## Abstract

Climate security refers to the risks posed by climate change on nations, societies, and individuals, including the possibility of conflicts. As an emerging field of research and public debate, where conceptual definitions are not yet fully agreed upon, gaining insights into global discussions on climate security enables systematizing its various interpretations and framings, mapping thematic priorities, and understanding information gaps that need to be filled. Considering Twitter as an important digital forum for information exchanges and dialogue, the dataset was created through the development of a query strategy based on a snowball scraping technique, which collected tweets containing hashtags related to climate security between January 2014 to May 2023. The dataset comprises 636,379 tweets. Content analysis was performed using text mining and network analysis techniques to generate additional data on sentiment, countries mentioned in the body of tweets, and hashtag co-occurrences. With almost 10 years of data, the utility of this dataset lies in the ability to assess the discursive evolution of a particular topic since its inception.

Specifications Table

**Table:** 

Subject	Social sciences
Specific subject area	Climate Security is an emerging multi-disciplinary field that investigates the complex interlinkages between climate, peace and security.
Data format	Mixed (raw and analysed)
Type of data	Database (.csv files) and social network connectivity (.gexf and .csv files).
Data collection	We used the open-source data collection tool 4CAT [1] to access the Twitter API and extract 636,379 tweets related to climate security from 01/01/2014 to 31/05/2023. Search queries included the expression ‘climate security’ in English, Spanish, and French, as well as 92 related hashtags. Additionally, text mining, sentiment analysis, and network analysis were performed to generate an annotated dataset and network data.
Data source location	Data was collected in Rio de Janeiro, Brazil, and is stored within the internal server of International Center for Tropical Agriculture (CIAT).
Data accessibility	Repository name: Zenodo Data identification number: 10.5281/zenodo.10198867 Direct URL to data: https://zenodo.org/records/10198867

## 1 Value of the Data

• As the climate crisis intensifies, the intersection of climate change with peace and security has become a fundamental area of concern, not only within academic circles but also among policymakers and civil society. In response, the field of Climate Security has emerged and evolved to explore these impacts through a human security lens.
• This dataset, encompassing nearly a decade of Twitter conversations about Climate Security, is pioneering in its scope and depth. Originating from a critical milestone when Climate Security first captured global attention, it offers useful insights into the evolution of public discourse on this essential topic.
• According to our understanding, this is the first publicly available dataset that offers a comprehensive archive of social media discussions on Climate Security. Unlike other datasets that might focus on climate change more generally, ours specifically concentrates on the interplay between global security and climate issues, providing a rich strand of data that captures a wide spectrum of public sentiment and geopolitical nuances. The inclusion of detailed annotations such as sentiment analysis and geographical tagging further enriches this dataset, enabling researchers to dissect the emotional and regional dimensions of the dialogue.
• The dataset utility extends beyond climate and security research, offering valuable resources for scholars in peace studies, conflict resolution, and policy formulation. By hydrating the tweets, researchers can perform a variety of analyses-ranging from trend assessments to complex network analyses. This allows for a deeper understanding of who are the key actors driving the agenda, how public conversations around Climate Security evolve, how they intersect with other major topics, and how perceptions shift in response to global events. Furthermore, policymakers can leverage insights from this dataset to gauge public opinion trends and craft more responsive and informed Climate Security strategies.
• Leveraging Twitter's hashtag feature, a key affordance that allows users to aggregate content by topic, this dataset provides a graph file describing the complex network structure and dynamics of the hashtags used in climate security tweets. This shows a living map of how Climate Security discussions relate to other key issues over time.
• The dataset aids in the systematic exploration of climate security narratives and supports the identification of thematic priorities, which could guide future research agendas and policy initiatives.

## 2 Background

``Climate security'' refers to risks caused by climate change that affects nations, societies, and individuals, including the possibility for conflicts. As a research field, climate security has evolved in the last 15 years from the margins of academic and policy circles to gain increased attention by the international community [2]. While the debate has grown across research, policy, and practice, the lack of a consensual definition of climate security raises the importance of monitoring global discussions and framings around the topic.

Relying on the digital methods epistemology, which leverages social media platforms as proxies for wider public discourse and engagement [3], and considering Twitter as an important digital forum for information exchanges and dialogue [4], a historical thematic dataset was collected to enable systematic exploration of the evolving narratives around climate security. Data is available from 2014, when the Intergovernmental Panel on Climate Change (IPCC) first included a chapter on human security in its Fifth Assessment Report [5], marking a key milestone in bringing climate security to the global arena. The temporal scale enables identifying and drilling down on topics, places, degrees of engagement and sentiments related to an important emerging discussion.

## 3 Data Description

All the data files are publicly available in Zenodo [6]. The dataset comprises 636,379 tweets (original tweets, retweets, quotes, or replies) related to climate security and tweeted between 01 Jan. 2014 and 31 May 2023. The repository hosts:

• A csv file containing the id of 636,379 tweets related to climate security: ‘ids_cs_tweets.csv';
• A txt file containing the queries used to filter data from the Twitter API (Table 1): ‘cs_twitter_queries.txt';
• A csv file containing the id, the date, the hashtags, the sentiment analysis result, and the list of mentioned countries for 636,379 tweets related to climate security: ‘cs_tweets_annotated.csv'
• A dynamic co-hashtags network file: ‘CS-co-hashtags-net_dynamic_GRAPH.gexf';
• A nodes table csv file consisting of 59,979 nodes from the dynamic co-hashtags network: ‘CS-co-hashtags-net_dynamic_NODES.csv';
• An edges table csv file consisting of 547,161 edges from the dynamic co-hashtags network: ‘CS-co-hashtags-net_dynamic_EDGES.csv';
• A pdf file containing the description of the data and instructions to employ a tool to hydrate Twitter data: readme.pdf.

## 4 Experimental Design, Materials and Methods

### 4.1 Dataset creation

This dataset was generated through a query strategy leveraging the snowball scraping technique. The expression “Climate security” in English, French, and Spanish and their hashtag equivalents — ``climate security'', ``securite climatique'', ``seguridad climatica'', ``#climatesecurity'', ``#securiteclimatique'', ``#seguridadclimatica'', — served as the starting points.

Our primary data extraction employed the 4CAT tool (Peeters & Hagen, 2021) to access the academic Twitter API v.2 and filter for tweets containing at least one climate security related keyword. We processed the initial tweets to extract the hashtags in order to proceed with the snowballing process. This phase involved a qualitative analysis of hashtags that occurred at least twice to assess their relation to climate security. 54 unique hashtags relevant to the topic were identified. Subsequently, a second scraping process was conducted to include the 54 hashtags to the initial list and expand the dataset. It entailed making three separate requests (see the queries listed in Table 1) to the Twitter API v.2 via 4CAT (Peeters & Hagen, 2021), constrained by the API's query limits of 500 characters.

The dataset's temporal scope starts in 2014, aligning with the publication of the IPCC's Fifth Assessment Report [5]. Firstly, data collection spanned from 1 January 2014 to 30 September 2022, with post-processing steps like merging query results and removing duplicates. A second snowballing step was conducted on 1 October 2022. This phase included an extended set of 38 new hashtags, culminating in a total of 92 hashtags contemplated in the query (see Table 1) and resulting in a dataset of 392,206 tweets. Data replenishments were conducted monthly until 31 May 2023, where exported CSV files were aggregated to the main dataset in R to generate the complete dataset of 636,379 tweets.

**Table 1:** Climate security Twitter queries.

query	text
1	#climatesecurity OR "climate security" OR #securiteclimatique OR "securite climatique" OR #seguridadclimatica OR "seguridad climatica" OR #weatheringrisk OR #sustainingpeace OR #environmentalsecurity OR #environmentalpeacebuilding OR #climatediplomacy OR #genderclimatesecurity OR #seguridadclimática OR #sécuritéclimatique OR #climadiplo OR #climateandsecurity OR #climatesecuritynexus OR #climatesecuritymechanism OR #climatediplopod OR #climatesecurity2017 OR #ecologicalsecurity OR #climateconflict OR #climateconflictnexus OR #climatepeacesecurity OR #climateampsecurity OR #climatefragility OR #climatesecurity06 OR #climatesecuritythe OR #waterconflict OR #euclimatediplomacy OR #climatesecurityisnationalsecurity OR #climatesecuritypractices OR #arcticclimatesecurity OR #climatesecuritygwu OR #climatesecurityhub OR #envconflictday OR #fpclimatesecurity OR #resourcesecurity OR #seguridadclimática
2	#sustainablepeace OR #aspclimatesecurity OR #climatefinanceforpeace OR #climateinsecurity OR #climatesecurity2020 OR #climatesecurityact OR climatesecuritypanel OR #climatesecurityrisks OR #climatesecuritys OR #climateterrorism OR #environmantalsecurity OR #environmentalconflict OR #environmentofpeace OR #resourceconflict OR #climatejustice OR #promotingsustainablepeace OR #environmentaljustice OR #naturesecurity OR #waterdiplomacy OR #climate_security OR #climatizedperspectiveonsecurity OR #cllimatejustice OR #adaptivepeace OR #climatejusticenow OR #naturesecuritynexus OR #waterpeacesec
3	#climatepeacenexus OR #greendefence OR #climateagrarianjustice OR #climatediplomacyweek OR #climatemigration OR #climatesecuriry OR #climateterrorismnexus OR #conflictclimate OR #ecosystemforpeace OR #planetarysecurity OR #unowasclimatepeacesecurity OR #africanclimatejustice OR #climate4peace OR #climatediplo OR #climatemobility OR #climatepeace OR #environmentalpeacemaking OR #environmentforpeace OR #environmentjustice OR #greendefense OR #naturalresourceconflicts OR #peaceandsustainability OR #sustainpeace OR #waterconflicts OR #waterdiplomay OR #wiistalksclimatesecurity OR #climate_terrorism

### 4.2 Data processing

The data handling was conducted using R, a statistical computing language, with the inclusion of specific packages to facilitate data manipulation and export. The data.table [7] package was employed for its advanced data manipulation capabilities, and writexl [8] was utilized for exporting the hashtags file to an Excel format for the creation of the dynamic network.

The climate security dataset was imported into R and tweet IDs were extracted and organized into a separate data table object, ‘dataset_CS_IDs', with the column name standardized to ``id''. This ID-specific data table was then exported as a CSV file, ``ids_cs_tweets.csv'', via the fwrite function for subsequent upload into the Zenodo repository.

To facilitate temporal analysis, the timestamp for each tweet was converted into a year-month-date format to remove the time information. Following this, a new data table, hashtags_net, was created to encapsulate the relationship between hashtags used in the tweets and the respective date of their tweeting. Finally, the hashtags_net data table was exported to an Excel file named ‘cs_hashtags_net.xlsx'. This file format was chosen to enable the use of Gephi [9] to generate the co-hashtags network file and export the nodes and edges tables.

To identify any countries mentioned in tweets, we developed an algorithm to search the bodies of text for mentions of the names of 249 countries in English, Spanish, French, Arabic, and the country's official major language, as well as country capital cities in English. The algorithm was developed in R using the data science collection of packages Tidyverse [10] and the package stringr [11] to manipulate individual characters within the strings in character vectors. It returns the ISO–3 country code for every country mentioned in a tweet, separated by commas.

To evaluate the sentiment of the tweets, we used the NRC lexicon NRC lexicon [13], a dictionary of words annotated with positive and negative sentiments that have translations in 108 languages. Using the get_sentiment function of the R package Syuzhet [12], we computed the sentiment for all the tweets written in Latin languages, which are supported by the package.

The final step was to create the annotated climate security Twitter dataset. Information about tweet IDs, date, hashtags, was merged by tweet ID in a data frame that was exported to a csv file (cs_tweets_annotated.csv) and uploaded in Zenodo.

## Limitations

This dataset has the following limitations. Firstly, the initial query was based only on English, Spanish, and French expressions and hashtags. While hashtags in English are often used in tweets written in other languages, this limits the dataset in terms of content and geographic coverage. Tweets generated in the Global North might be overrepresented in relation to content in languages that do not use the Latin alphabet. Additionally, we must consider limitations in accurately understanding the nuances and complexities of human emotions through algorithmic interpretation. Although the NRC Emotion Lexicon used in the sentiment analysis provides versions of the lexicon in 108 languages, it can express flaws in capturing context, irony, sarcasm, and subtle linguistic cues. Consequently, some interpretations might be incorrect or out of context. Furthermore, our country detection algorithm does not include sub-national location names or places, which limits the ability to identify countries when other geographical identifiers are utilized. Lastly, data collection was interrupted in May 2023 as Twitter's Academic API was shut down.

## Ethics Statement

To align with Twitter's Developer Agreement and Policy [14], this dataset does not include the actual content of tweets. Instead, it provides tweet IDs and associated annotations for further analysis. The dataset does not contain any identifiable information related to Twitter profiles, ensuring privacy and compliance with data protection guidelines.

For reproducibility purposes, we indicate an appropriate hydration tool that allows recovering tweets content and metadata from ID by accessing the Twitter API.

## CRediT authorship contribution statement

**Bia Carneiro:** Conceptualization, Methodology, Validation, Writing – review & editing, Project administration. **Giulia Tucci:** Methodology, Software, Data curation, Writing – review & editing.

## Data Availability

Climate Security on social media: raw and processed data from Twitter (Original data) (Zenodo)
